# Abdominal ectopic pregnancy – misdiagnosed upper abdominal pain: A case report

**DOI:** 10.1097/MD.0000000000048484

**Published:** 2026-04-24

**Authors:** Hong Xu, Xiaohong Xie, Caihong Wu

**Affiliations:** a Department of Gynecology, Suzhou Ninth People’s Hospital, Suzhou Ninth Hospital Affiliated to Soochow University, Suzhou, China; b Department of Surgery, Suzhou Ninth People’s Hospital, Suzhou Ninth Hospital Affiliated to Soochow University, Suzhou, China.

**Keywords:** abdominal ectopic pregnancy, case report, laparoscopic surgery, surgical management

## Abstract

**Rationale:**

Ectopic pregnancy (EP) with implantation in the upper abdomen is exceptionally rare. It represents a challenging clinical scenario with very limited evidence confined to case reports. It seriously endangers maternal health and early diagnosis and treatment can significantly improve patient prognosis.

**Patient concerns:**

We report a 25-year-old woman who was diagnosed several days ago with gastroenteritis was referred to our department because of worsened upper left abdominal pain. Contrast computerized tomography scan revealed a mass measuring ~46 mm × 40 mm × 56 mm in the upper left abdomen and her serum human chorionic gonadotropin level was 10,130.28 IU/L. EP was highly suspected on admission.

**Diagnoses:**

Abdominal EP was confirmed by intraoperative findings and postoperative pathology.

**Interventions:**

The patient underwent laparoscopic surgery which went smoothly after admission. An gastrointestinal surgeon was involved during the surgery and the excision of the EP lesion along with partial omentum was performed.

**Outcomes:**

The patient was discharged 5 days later after operation without complications. The serum human chorionic gonadotropin level declined to normal within 1 month follow-up.

**Lessons:**

This case illustrates that although ultrasound is considered the first-line method for diagnosing EP, abdominal lesions with undefined nature detected by computerized tomography should also be pay attention to, especially for women of childbearing age presenting with gastrointestinal symptoms other than typical ones such as lower abdominal pain or vaginal bleeding. Close monitoring and timely management are pivotal to improve patient prognosis.

## 1. Introduction

An ectopic pregnancy (EP) is defined as the developing products of conception implant outside the endometrial cavity with tubal EP accounting for almost 95% of all.^[1]^ The abdominal cavity is a rare site of ectopic implantation, occurring in approximately 1% of EP, especially for primary ones.^[2,3]^ Management of such rare conditions is challenging because they often induce organ perforation and life-threatening maternal hemorrhage.^[4]^ Herein, we report a case of a 25-year-old female who was transferred to our emergency department with upper left abdominal pain accompanied by intermittent nausea and vomiting, abdominal ectopic pregnancy (AEP) was highly suspected by computerized tomography (CT) images and elevated serum human chorionic gonadotropin (HCG) level, the patient underwent diagnostic laparoscopy at last and discharged without complications. An AEP was diagnosed by surgical and pathological findings. Rapid and accurate diagnosis pre-operatively is critical for improving the prognosis of a child bearing woman with an abdominal pregnancy.

## 2. Case report

**Figure 1.**
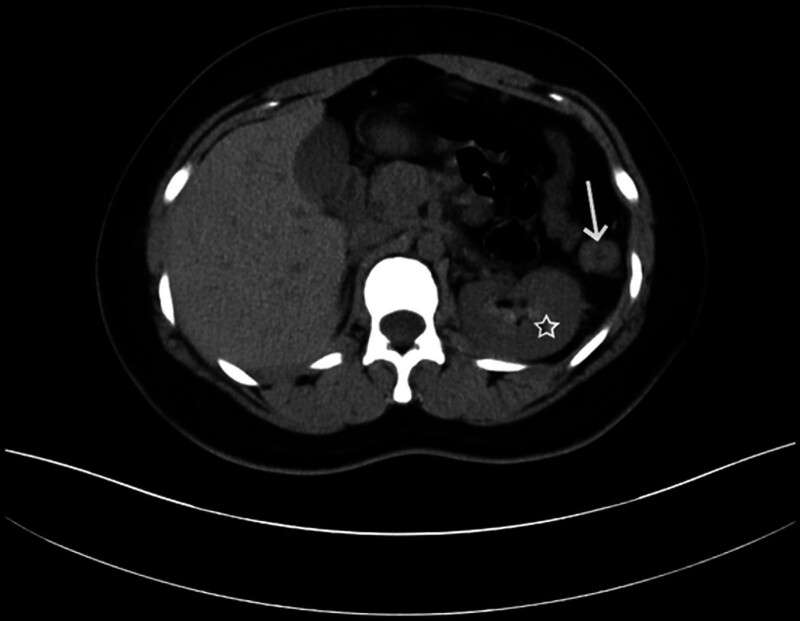
Non-contrast CT showing the lesion in the left upper abdomen ~21 mm × 19 mm × 10 mm (white arrow indicated the lesion and ☆indicated the left kidney). CT = computerized tomography.

A 25-year-old woman (gravida 1, parity 0) at approximately 5 weeks of pregnancy was referred to our emergency department on April 7, 2025 due to pain in the upper left side of her abdomen along with intermittent vomiting and nausea. The female had a regular 28 to 30 days menstrual cycle and her last menstrual period was on March 3rd. On March 30th, the patient visited the local community hospital due to mild left upper abdominal pain. A non-contrast CT scan revealed a mass with undetermined nature in the left upper abdomen, measuring ~21 mm × 19 mm × 10 mm (Fig. 1). Because of the unavailability of contrast CT, the hospital was unable to perform further examination on the patient. She was initially diagnosed with gastroenteritis and treated with anti-inflammatory, acid-suppressing, and antispasmodic medications for 1 week, but her pain did not alleviate.

**Figure 2.**
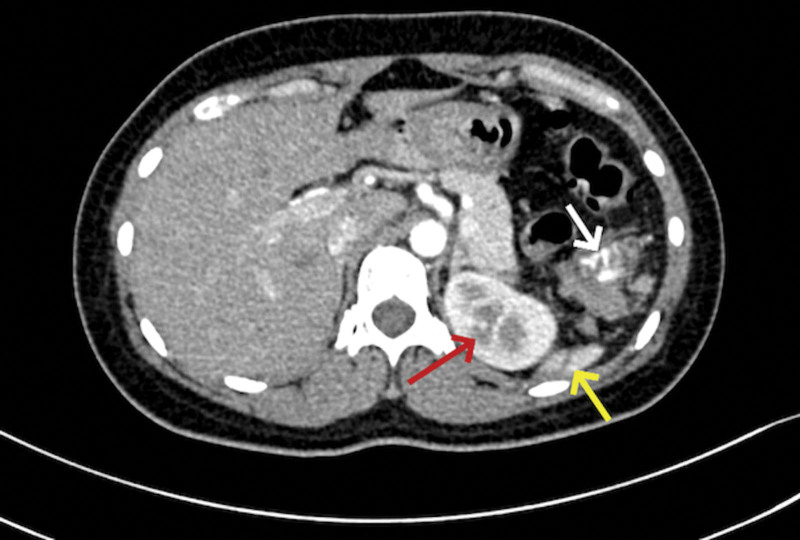
Contrast CT (arterial phase) showing the lesion at the same location measuring ~46 mm × 40 mm × 56 mm, it was surrounded by blood vessels (white arrow indicated the lesion; yellow arrow indicated the spleen; red arrow indicated the left kidney). CT = computerized tomography.

On April 6th, the patient self-tested positive for urinary pregnancy due to amenorrhea. She experienced worsened left upper abdominal pain on the next day, accompanied by nausea and vomiting. She was transferred to our hospital and an emergency ultrasound was conducted: no gestational sac was visualized within the uterine cavity, endometrial thickness measured 8 mm, no abnormalities were detected in the bilateral adnexal regions. A much more larger mass at the same location measuring ~46 mm × 40 mm × 56 mm, which was surrounded by blood vessels was revealed via contrast CT (Fig. 2). Considering the patient’s medical history, she was admitted to the gynecology department for further assessment with primary consideration of EP.

**Figure 3.**
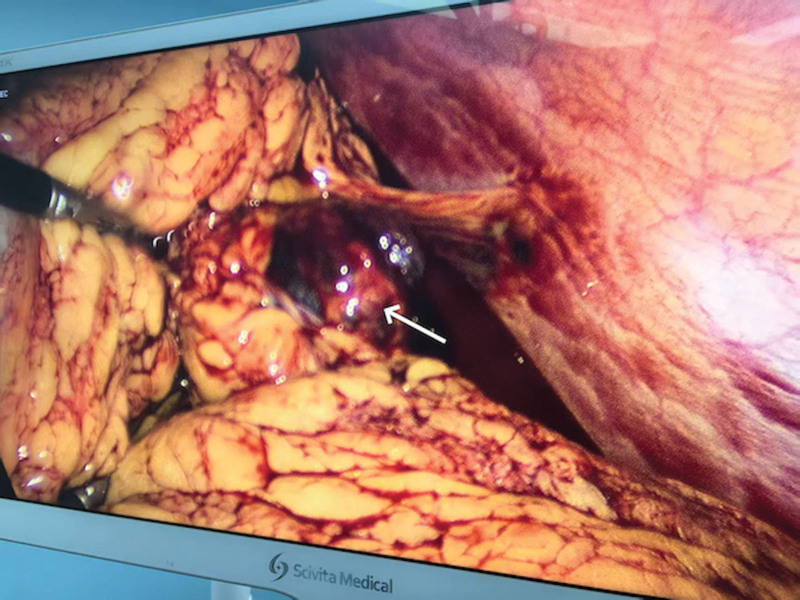
Intraoperative finding showing a lesion measuring 6 cm × 4 cm × 5 cm was identified where the products of conception were detected (white arrow).

**Figure 4.**
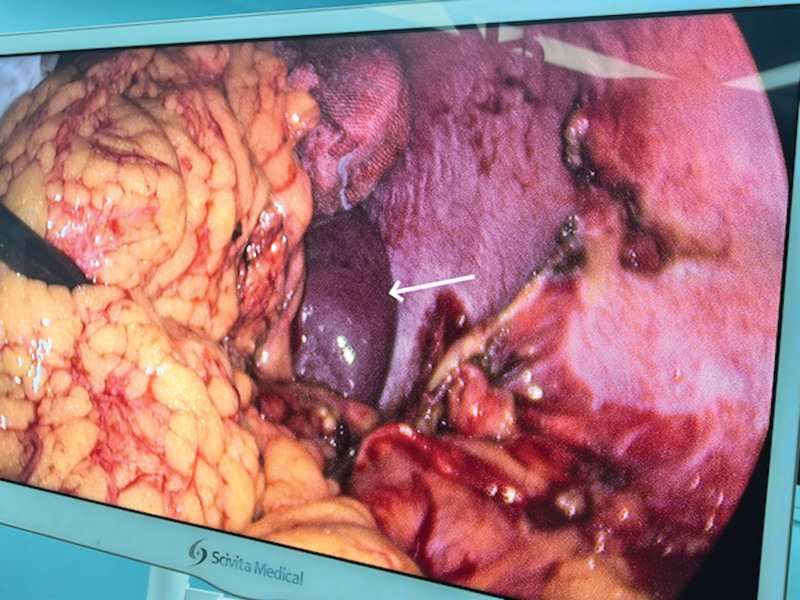
During the surgery we can see the edge of the spleen (white arrow) after removing the lesion.

**Figure 5.**
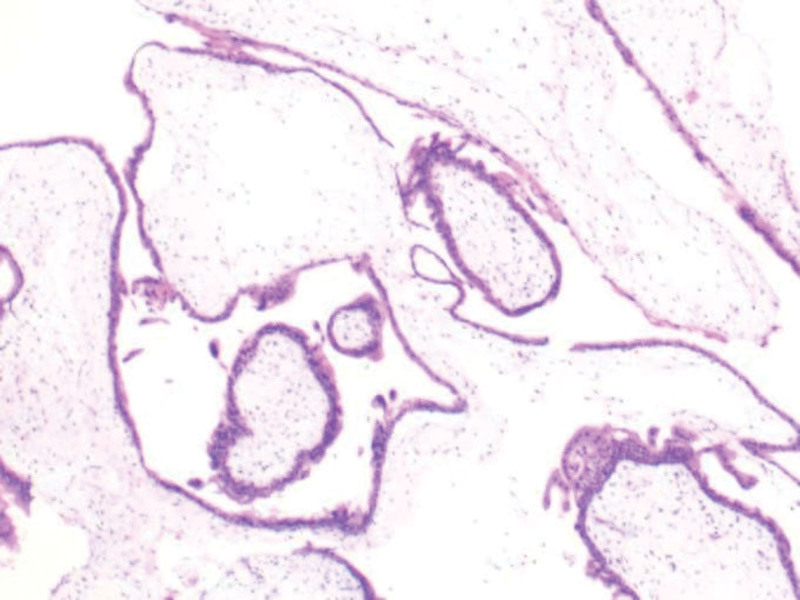
Microscopic findings using hematoxylin and eosin (HE) staining, We identified the villous tissue on the image. HE = hematoxylin and eosin.

**Figure 6.**
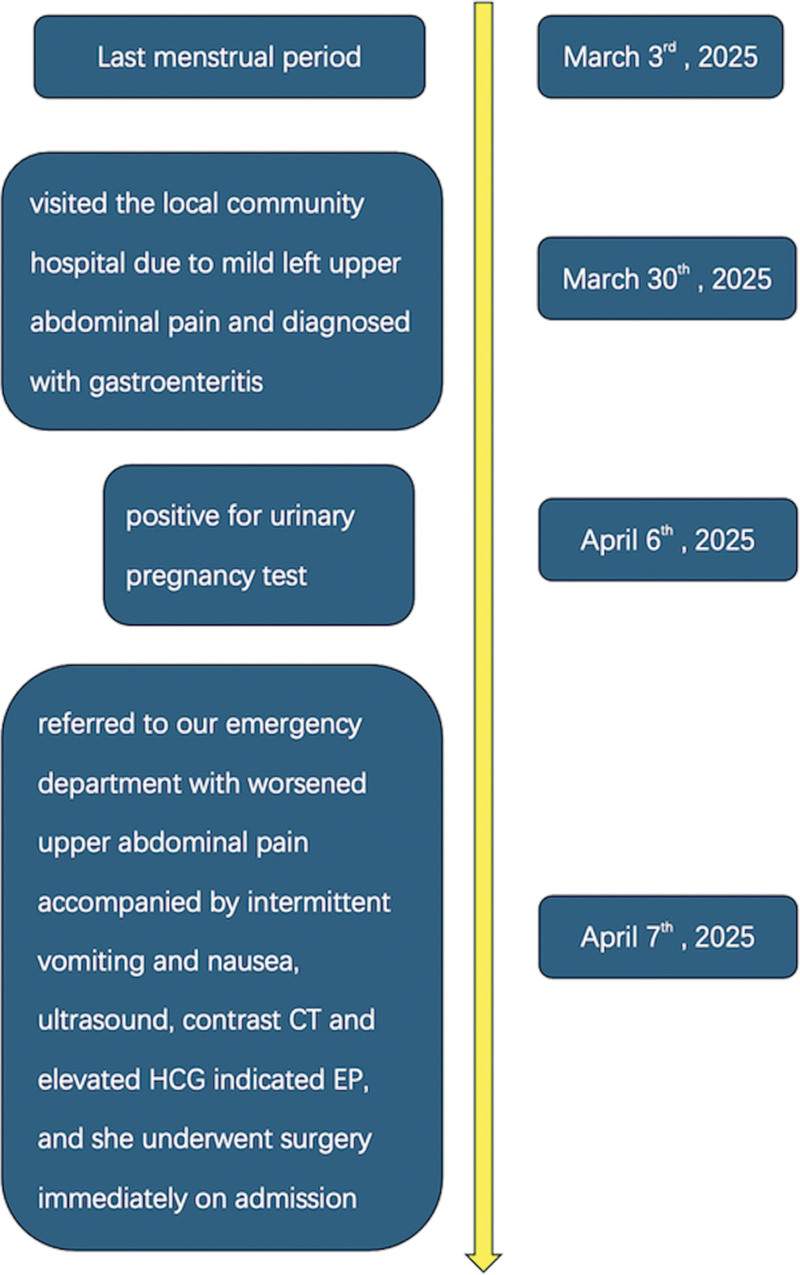
Timeline of the entire diagnostic and therapeutic process.

**Figure 7.**
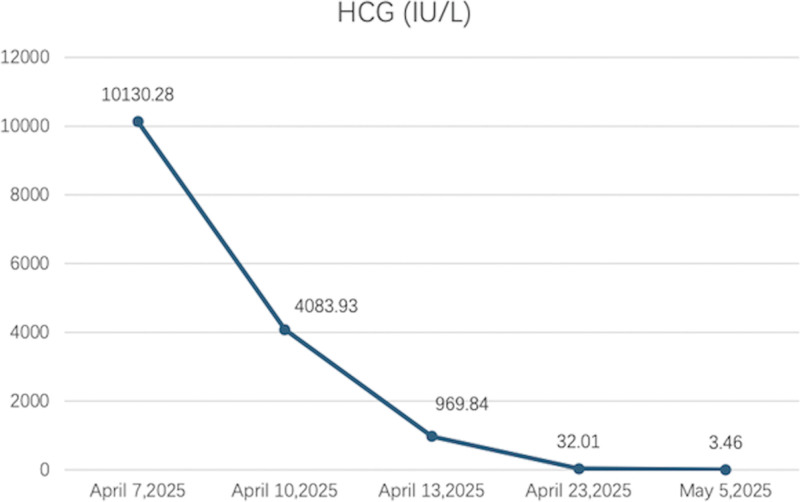
The change in HCG during the process. HCG = human chorionic gonadotropin.

The initial vital signs were stable on admission: systolic and diastolic blood pressure were 107 mm Hg and 77 mm Hg, respectively. The initial pulse rate was 89 beats per minute and temperature was 36.7°C. Clinical examination identified left upper abdominal pain with localized tenderness. No tenderness, guarding or rigidity was noted in the lower abdomen. she had normal external female genitalia and no active vaginal bleeding; the cervix was closed. Upon bimanual examination there were no cervical motion tenderness and palpable adnexal mass. Blood tests showed the serum HCG level was 10,130.28 IU/L. She conceived naturally this time and had no history of pelvic inflammatory disease. She also denied history of chronic medical or surgical illnesses and reported no tobacco or alcohol consumption. Since magnetic resonance imaging (MRI) requires an appointment at our hospital and the patient could not afford the cost at a private facility. A diagnostic laparoscopy was performed under general anesthesia upon admission to confirm the suspicion of an EP, intraoperative findings were as follows: about 300 mL of hemoperitoneum was noted in the abdominopelvic cavity, a hematoma was visualized between the inferior border of the spleen and the descending colon, enveloped by the greater omentum with active bleeding. After aspiration of hemoperitoneal fluid, a lesion measuring 6 cm × 4 cm × 5 cm was identified where the products of conception were detected (Figs. 3 and 4). All other pelvic organs including uterus and bilateral ovaries and tubes appeared grossly normal in appearance. An gastrointestinal surgeon was involved during the surgery and the excision of the EP lesion along with partial omentum was performed. An AEP was confirmed by pathology finally (Fig. 5). The patient had an uneventful recovery and discharged 5 days after surgery. Her serum HCG level declined to normal rang (<5 IU/L) within 1 month follow-up. The entire diagnostic and therapeutic process and the change in HCG are summarized below (Figs. 6 and 7).

## 3. Discussion

EP is a term used to describe any implantation of a fertilized ovum outside the uterine cavity and an AEPis an embryo that implants on any structure within the peritoneal cavity.^[1,5]^ The most ectopic pregnancies occur in the fallopian tube (about 95.5%), 1.3% arise in abdominal locations.^[3]^ It is reported that the mortality rate of AEP is 7.7 times higher than that of tubal ones.^[6]^ Early diagnosis is difficult to establish as women may present asymptomatically and there is often lack of sufficient evidence to distinguish it from a tubal pregnancy, Chen et al reviewed 17 cases of abdominal pregnancy which showed only 29.41% (5/17) of patients were diagnosed before surgery, and the other 70.59% (12/17) were misdiagnosed with tubal pregnancy or unexplained intra-abdominal hemorrhage.^[7]^ Ruptured EPs are the leading cause of maternal death within the first trimester of pregnancy and account for approximately 5% to 10% of all pregnancy-related deaths^[1]^

AEP is classified into 2 types: either primary (the blastocyst is directly implanted on the surface of the peritoneum or the viscera of the abdominal cavity in the early stages of pregnancy) or secondary (the embryo falls from the fallopian tube into the abdominal cavity).^[8]^ Cases which meet the following criteria are defined as primary ones: tubes and ovaries with normal appearance, no evidence of an utero-peritoneal fistula, products conception only exist in the abdominal cavity and early enough in gestation to eliminate the possibility of secondary implantation after the primary nidation of the tube.^[9]^ In our case, the patient initially visited the hospital on day 28 of her cycle due to upper abdominal pain, and a solitary lesion in the left upper abdomen was observed at that time via CT. During the surgery, no abnormalities were observed in the uterus and bilateral adnexal regions and she was confirmed to have an EP at 5 weeks of gestation. According to the above information, the patient was considered to have a primary AEP.

Compared with tubal pregnancies, vaginal bleeding is less common in AEP,^[10]^ resulting in further delay in the diagnosis of AEP. As in our case, not until the patient had a history of amenorrhea and a positive urinary pregnancy test did we consider the diagnosis of an EP. Lack of specific symptoms makes diagnosis and management challenging. Additionally, risk factors associated with EP mentioned below should be keep in mind: age >35 years; cigarette smoking; documented fallopian tube pathology; infertility; pelvic inflammatory disease; pregnancy while IUD in place; previous EP; previous fallopian tube surgery; the use of ART.^[4,11]^ A careful inquiry into the history of a patient is of great importance for the diagnosis of EP.

Current diagnostic standard for EP includes transvaginal ultrasound combined with serum HCG monitoring. There is high suspicion for EP when serum HCG level of a patient is >2000 IU/L with no sign of intrauterine pregnancy.^[1,4]^ Ultrasound, especially transvaginal ultrasound, remains the first-line protocol for diagnosing EP^[10]^ MRI can be a useful diagnostic adjunct in advanced abdominal pregnancy and can help to plan the surgical approach.^[12]^ In our case, since MRI was not available, we performed contrast CT as an alternative method which pointed toward AEP. For women of reproductive age, HCG detection is essential if the patient complained of abnormal vaginal bleeding in case of misdiagnosis of EP. However, with the application of ART, which increases the incidence of EP,^[13]^ there were exceptional cases where patients with very low level of HCG were diagnosed with AEP,^[14-16]^ therefore, even if a woman’s HCG level is within normal range, there is a certain possibility of an AEP when the patient has symptoms such as abdominal pain or vaginal bleeding especially she conceived through ART.

The management options for EPs are usually divided into 3 categories: expectant, medical and surgical. As far as our knowledge extends, there exists no standardized therapeutic protocol for AEP. Although there were selected patients who recovered well by MTX treatment,^[17]^ surgical resection was considered to be the standard treatment for abdominal pregnancy and laparoscopic removal is an optimized approach compared to laparotomy for treatment of early abdominal pregnancy.^[4,12]^ However, if there is uncontrollable massive bleeding, conversion to laparotomy should be required to prevent maternal death. In this case, expectant and medical therapy was not recommended since active bleeding existed in the abdominal cavity, we completely resected the lesion under laparoscopy, but if the serum level of HCG were not to decline to normal postoperatively, it would be necessary to use MTX to eradicate remaining trophoblastic cells.

At present, it is believed that abdominal pregnancy is the only type of EP that can advance beyond 20 weeks of gestational age.^[2]^ At late gestations, surgery is the main treatment, the key issue is how to manage the placenta. It may lead to exacerbated bleeding if proper control of the vascular supply cannot be achieved. In some cases, clinicians use vascular embolization to minimize intraoperative bleeding and completely remove the placenta; other treatment methods include leaving the placenta in situ and waiting for absorption either naturally or by use of MTX; besides, partial removal of the placenta and surgical excision of the remaining part 12 days postoperatively was also reported.^[2,7,18,19]^ In conclusion, disposal of the placenta requires more evidence-based medicine and a good separation technique and adequate vascular management should be performed.

## 4. Conclusion

This report describes the manifestation, diagnosis, and treatment of a rare clinical condition. For this case, sufficient communication should be made with the patient before surgery, and the gestational sac implantation site should be evaluated through various examinations to improve diagnostic accuracy. Lack of experience of the radiographer and the patient’s initial presentation with upper abdominal pain made early diagnosis tougher. Management of complicated EPs requires a multidisciplinary team involving experienced obstetricians, diagnostic radiologists and general surgeons to reduce surgical risks and prevent adverse maternal outcomes.

## Author contributions

**Data curation:** Hong Xu.

**Funding acquisition:** Hong Xu.

**Investigation:** Xiaohong Xie, Caihong Wu.

**Resources:** Hong Xu, Xiaohong Xie, Caihong Wu.

**Supervision:** Caihong Wu.

**Writing – original draft:** Hong Xu.

**Writing – review & editing:** Xiaohong Xie, Caihong Wu.

## References

[1] Houser M Kandalaft N Khati NJ. Ectopic pregnancy: a resident’s guide to imaging findings and diagnostic pitfalls. Emerg Radiol. 2022;29:161–72. 34618256 10.1007/s10140-021-01974-7

[2] Dunphy L Boyle S Cassim N Swaminathan A. Abdominal ectopic pregnancy. BMJ Case Rep. 2023;16:e252960. 10.1136/bcr-2022-252960 PMC10546113 37775278

[3] Kang OJ Koh JH Yoo JE. Ruptured hemorrhagic ectopic pregnancy implanted in the diaphragm: a rare case report and brief literature review. Diagnostics (Basel). 2021;11:2342. 34943579 10.3390/diagnostics11122342 PMC8699918

[4] Mullany K Minneci M Monjazeb R Coiado OC. Overview of ectopic pregnancy diagnosis, management, and innovation. Womens Health (Lond). 2023;19:17455057231160349. 36999281 10.1177/17455057231160349 PMC10071153

[5] Chong KY de Waard L Oza M. Ectopic pregnancy. Nat Rev Dis Primers. 2024;10:94. 39668167 10.1038/s41572-024-00579-x

[6] Ishikawa Y Nakanishi K Tsumura A Murakami K Nishiwaki K. Early abdominal wall ectopic pregnancy treated with laparoscopic surgery: a case report and literature review. J Obstet Gynaecol Res. 2023;49:2544–8. 37424208 10.1111/jog.15739

[7] Chen Y Peng P Li C. Abdominal pregnancy: a case report and review of 17 cases. Arch Gynecol Obstet. 2023;307:263–74. 35474494 10.1007/s00404-022-06570-9 PMC9837172

[8] Zheng X Zhou Y Sun Z Yan T Yang Y Wang R. Abdominal pregnancy secondary to uterine horn pregnancy: a case report. BMC Pregnancy Childbirth. 2023;23:412. 37270533 10.1186/s12884-023-05704-4 PMC10239574

[9] Studdiford WE. Primary peritoneal pregnancy. Am J Obstet Gynecol. 1942;44:487–91.

[10] Tegene D Nesha S Gizaw B Befikadu T. Laparotomy for advanced abdominal ectopic pregnancy. Case Rep Obstet Gynecol. 2022;2022:3177810. 35299756 10.1155/2022/3177810 PMC8923797

[11] Hendriks E Rosenberg R Prine L. Ectopic pregnancy: diagnosis and management. Am Fam Physician. 2020;101:599–606. 32412215

[12] Elson CJ Salim R Potdar N Chetty M Ross JA Kirk EJ. Diagnosis and management of ectopic pregnancy. BJOG. 2016;123:e15–55.27813249

[13] Yoder N Tal R Martin JR. Abdominal ectopic pregnancy after in vitro fertilization and single embryo transfer: a case report and systematic review. Reprod Biol Endocrinol. 2016;14:69. 27760569 10.1186/s12958-016-0201-x PMC5070159

[14] Li Y Geng J He Q. Abdominal ectopic pregnancy following a frozen embryo transfer cycle: a case report. BMC Pregnancy Childbirth. 2021;21:707. 34674658 10.1186/s12884-021-04133-5 PMC8532271

[15] Irani M Elias RT Pereira N Gunnala V Rosenwaks Z. Abdominal ectopic pregnancy with undetectable serum β-human chorionic gonadotropin 9 days following blastocyst transfer. J Obstet Gynaecol Res. 2016;42:1886–8. 27718286 10.1111/jog.13127

[16] Yanaihara A Ohgi S Motomura K. An abdominal ectopic pregnancy following a frozen-thawed ART cycle: a case report and review of the literature. BMC Pregnancy Childbirth. 2017;17:108. 28388882 10.1186/s12884-017-1294-8 PMC5383944

[17] Beck MH Sehouli J Leppig JA Knitter S Pratschke J Krenzien F. Multimodal management of ectopic hepatic pregnancy: a systematic review of the literature. Arch Gynecol Obstet. 2024;310:2345–53. 39352540 10.1007/s00404-024-07739-0 PMC11485115

[18] Wu Q Bai S Han L Song L. Secondary mid-term abdominal pregnancy: a case report. Medicine (Baltim). 2025;104:e43281. 10.1097/MD.0000000000043281 PMC12282748 40696617

[19] Gorincour G Boukerrou M. Abdominal ectopic pregnancy. N Engl J Med. 2023;389:e51. 38078501 10.1056/NEJMicm2120220

